# Knockdown of long noncoding RNA HOTAIR inhibits osteoarthritis chondrocyte injury by miR-107/CXCL12 axis

**DOI:** 10.1186/s13018-021-02547-7

**Published:** 2021-06-28

**Authors:** Jipeng Lu, Zhongxiong Wu, Ying Xiong

**Affiliations:** Department of Orthopedics, Yan’an Hospital Affiliated to Kunming Medical University, No. 245 Renmin East Road, Panlong District, Kunming, 650051 Yunnan China

**Keywords:** Osteoarthritis, Chondrocyte, HOTAIR, miR-107, CXCL12

## Abstract

**Background:**

Osteoarthritis (OA) is a joint disease characterized via destruction of cartilage. Chondrocyte damage is associated with cartilage destruction during OA. Long noncoding RNAs (lncRNAs) are implicated in the regulation of chondrocyte damage in OA progression. This study aims to investigate the role and underlying mechanism of lncRNA homeobox antisense intergenic RNA (HOTAIR) in OA chondrocyte injury.

**Methods:**

Twenty-three OA patients and healthy controls without OA were recruited. Chondrocytes were isolated from OA cartilage tissues. HOTAIR, microRNA-107 (miR-107) and C-X-C motif chemokine ligand 12 (CXCL12) levels were measured by quantitative real-time polymerase chain reaction and western blot. Cell proliferation, apoptosis and extracellular matrix (ECM) degradation were measured using cell counting kit-8, flow cytometry and western blot. The target interaction was explored by bioinformatics, luciferase reporter and RNA immunoprecipitation assays.

**Results:**

HOTAIR expression was enhanced, and miR-107 level was reduced in OA cartilage samples. HOTAIR overexpression inhibited cell proliferation, but induced cell apoptosis and ECM degradation in chondrocytes. HOTAIR knockdown caused an opposite effect. MiR-107 was sponged and inhibited via HOTAIR, and knockdown of miR-107 mitigated the effect of HOTAIR silence on chondrocyte injury. CXCL12 was targeted by miR-107. CXCL12 overexpression attenuated the roles of miR-107 overexpression or HOTAIR knockdown in the proliferation, apoptosis and ECM degradation. CXCL12 expression was decreased by HOTAIR silence, and restored by knockdown of miR-107.

**Conclusion:**

HOTAIR knockdown promoted chondrocyte proliferation, but inhibited cell apoptosis and ECM degradation in OA chondrocytes by regulating the miR-107/CXCL12 axis.

**Supplementary Information:**

The online version contains supplementary material available at 10.1186/s13018-021-02547-7.

## Introduction

Osteoarthritis (OA) is one of the most common joint diseases, which can involve all joint tissues, such as the subpatellar fat pad, synovial membrane, subchondral bone, meniscus, ligaments and cartilage [1, 2]. Chondrocytes are integral to the anabolic–catabolic balance in the cartilage, and their abnormalities are responsible for the destruction of cartilage during OA [3]. Chondrocyte is the only cell type of cartilage, and the apoptosis and extracellular matrix (ECM) degradation of chondrocytes are related to OA progression [4, 5]. Therefore, exploring the mechanism of chondrocyte injury might indicate new insight into the pathology of OA, and provide novel target for OA treatment.

Noncoding RNAs are involved in cartilage development and chondrocyte injury [6, 7]. Long noncoding RNAs (lncRNAs; with > 200 nucleotides in length) are a type of noncoding RNA that can be involved in cartilage development and degeneration, and have important values in the treatment of bone-related diseases, including OA [8, 9]. Moreover, lncRNAs play important roles in OA progression by regulating chondrocyte processes, like proliferation, apoptosis, inflammation and ECM [10]. LncRNA homeobox antisense intergenic RNA (HOTAIR) is implicated in multiple cell biological processes, including proliferation, migration, invasion and apoptosis by acting as a competitive endogenous RNA (ceRNA) through interacting with microRNAs (miRNAs) [11, 12]. A previous report revealed that HOTAIR could increase chondrocyte apoptosis which was regulated via lncRNA P50-associated cyclooxygenase-2 extragenic RNA (PACER) [13]. Furthermore, HOTAIR might promote OA progression by regulating ECM degradation and chondrocyte apoptosis through the miR-17-5p/fucosyltransferase 2 (FUT2)/β-catenin axis [14]. Nevertheless, the mechanisms are complex, and more regulatory networks mediated by HOTAIR in chondrocyte damage during OA progression are needed to be explored.

MiRNAs are a class of noncoding RNAs with ~ 22 nucleotides interacting with cognate mRNAs, which play key roles in OA progression [15]. A former study suggested that miR-107 could mitigate chondrocyte apoptosis and ECM degradation via regulating phosphatase and tensin homolog deleted on chromosome ten (PTEN) [16]. Zhao *et al*. reported that miR-107 could inhibit apoptosis and promote autophagy of chondrocytes during OA progression by regulating tumor necrosis factor receptor associated factor three (TRAF3) [17]. However, whether miR-107 is responsible for HOTAIR to regulate chondrocyte damage during OA remains unclear. Moreover, many differentially expressed genes are involved in the development of chondrogenesis during OA [18], in which C-X-C motif chemokine ligand 12 (CXCL12) has been reported to participate in the development of some inflammatory diseases [19]. Bioinformatics assay showed miR-107 could bind with HOTAIR and CXCL12. Thus, we hypothesized HOTAIR might regulate CXCL12 by competitively binding with miR-107. However, the HOTAIR/miR-107/CXCL12 axis has not been reported in OA progression.

In an attempt to elucidate the function and regulatory mechanism of HOTAIR in OA progression, this research focused on the effect on cell proliferation, apoptosis and ECM degradation in OA chondrocytes, and investigated the regulatory network of HOTAIR/miR-107/CXCL12.

## Materials and methods

### Cartilage tissues collection

Twenty-three OA knee cartilage samples were harvested from OA patients who underwent total knee arthroplasty. The normal knee cartilage samples were obtained from 23 patients with surgical repair of fractures, who did not have OA. The characteristics of all subjects are shown in Table 1. All patients recruited in this research have signed the informed consents. The study was approved by the ethics committee of Yan’an Hospital Affiliated to Kunming Medical University.

**Table 1:** Baseline characteristics of the study subjects

Characteristics	Normal (n = 23)	OA (n = 23)
Age		
> 60 years	12	13
≤ 60 years	11	10
Gender		
Male	10	9
Female	13	14
OA grading		
Grade I	—	3
Grade II	—	11
Grade III	—	8
Grade IV	—	1
BMI	24.6 ± 3.1	27.4 ± 2.7

*BMI* Body mass index

### Cell culture and transfection

Chondrocytes were isolated from knee cartilage tissues of 3 OA patients for 3 independent experiments using collagenase digestion method as previously reported [20, 21]. In brief, the inflammatory cartilage tissues were minced, digested with trypsin (Sigma-Aldrich, St. Louis, MO, USA) at 37 °C for 30 min, and then incubated with 0.2% collagenase II (Sigma-Aldrich) at 37 °C for 6 h. After centrifugation at 1500 g for 10 min, cells were maintained in DMEM (Thermo Fisher Scientific, Waltham, MA, USA) containing 10% fetal bovine serum at 37 °C under 5% CO_2_. The third passages of cells were used in this research.

Small interfering RNA (siRNA) against HOTAIR (si-HOTAIR), siRNA negative control (si-NC), pcDNA-based HOTAIR overexpression vector (HOTAIR), pcDNA-based CXCL12 overexpression vector (CXCL12), pcDNA empty vector, miR-107 mimic (miR-107), miRNA negative control (miR-NC), miR-107 inhibitor (in-miR-107) and inhibitor negative control (in-miR-NC) were synthesized from Genomeditech (Shanghai, China). The chondrocytes were transfected with 1 μg vectors or 40 nM oligos using Lipofectamine^TM^ 2000 Transfection Reagent (Thermo Fisher Scientific) for 6 h. After 24 h, chondrocytes were collected for subsequent experiments.

### Quantitative real-time polymerase chain reaction (qRT-PCR)

Total RNA was isolated using Trizol (Thermo Fisher Scientific). The reverse transcription was performed with 1 μg RNA using All-in-One^TM^ first strand cDNA synthesis kit (FulenGen, Guangzhou, China). The cDNA product was diluted by 1:10, and used for qRT-PCR with specific primers and SYBR Green (Vazyme, Nanjing, China). The qRT-PCR was conducted on CFX96™ Real-time PCR Detection System (Bio-Rad, Hercules, CA, USA). The primers used in this research were generated by Sangon Biotech (Shanghai, China) as follows: HOTAIR (forward, 5’-GTTTGGGATCTGTTCCAGCCT-3’; reverse, 5’-CGTCTGTAACTCTGGGCTCC-3’); CXCL12 (forward, 5’-CTACAGATGCCCATGCCGAT-3’; reverse, 5’-GTGGGTCTAGCGGAAAGTCC-3’); β-actin (forward, 5’-CTTCGCGGGCGACGAT-3’; and reverse, 5’-CCACATAGGAATCCTTCTGACC-3’). The primers of miR-107 and U6 were purchased from FulenGen (HmiRQP0030 and HmiRQP9001). Using β-actin or U6 as reference control, the relative RNA expression was analyzed via the 2^-ΔΔCt^ method [22].

### Cell proliferation assay

The cell counting kit-8 (CCK-8) kit (Solarbio, Beijing, China) was used for measurement of chondrocyte proliferation. Chondrocytes (3 × 10^3^ cells) were inoculated into 96-well plates in triplicate. Cells were cultured for 0, 24, 48 and 72 h, and then interacted with 10 μL CCK-8 solution for another 3 h. Subsequently, the optical density (OD) value at 450 nm of each well was measured using a microplate reader (Potenov, Beijing, China).

### Cell apoptosis assay

The detection of cell apoptosis was performed with Annexin V-FITC/PI apoptosis detection kit (Vazyme) through flow cytometry. Chondrocytes (5 × 10^4^ cells) were plated into 6-well plates and grown at 37 °C for 72 h. Then cells were collected and double stained with 10 μL Annexin V-FITC and PI under condition void of light for 10 min. The stained cells were measured with a NovoCyte 1040 flow cytometer (Agilent Biosciences, Hangzhou, China), and the apoptotic rate was expressed as the percentage of cells in the lower and upper right.

### Bioinformatics analysis and luciferase reporter assay

Bioinformatics analysis using DIANA tools was performed, and showed the binding sites of miR-107 and HOTAR or CXCL12 3’UTR. Based on pmirGLO vectors (Promega, Madison, WI, USA), the sequences of HOTAIR or CXCL12 3’ UTR with miR-107 binding sites or mutant sites was used to generate wild-type (WT) or mutant (MUT) luciferase reporter vectors, named as HOTAIR WT, HOTAIR MUT, CXCL12 3’UTR WT or CXCL12 3’UTR MUT, respectively. miR-107 mimic, miR-NC, in-miR-107 or in-miR-NC was co-transfected with the luciferase reporter vectors into chondrocytes using Lipofectamine^TM^ 2000 Transfection Reagent. Cells were collected at 24 h after the transfection, and the relative luciferase activity was detected with a luciferase reporter assay kit (Promega).

### RNA immunoprecipitation (RIP)

The RIP assay was conducted in chondrocytes, with the Magna RNA immunoprecipitation kit (Millipore, Billerica, MA, USA). Cells (1 × 10^7^) were lysed, and then incubated with RIP immunoprecipitation buffer containing magnetic beads pre-coated with Ago2 antibody for 8 h. IgG was regarded as control. The anti-Ago2 (ab32381) was purchased from Abcam (Cambridge, MA, USA), and anti-IgG (AP112) was obtained from Sigma-Aldrich. The level of HOTAIR enriched in complex was detected by qRT-PCR.

### Western blot

The cartilage tissues or chondrocytes were lysed using RIPA buffer with 1% PMSF (Beyotime) on ice. The BCA Protein Assay Kit (Beyotime) was applied to quantify protein, and then proteins were denaturalized by boiling water bath. The electrophoresis and membrane transfer were performed using Bio-Rad Bis-Tris Gel system (Bio-Rad) following the manufacturer’s instructions. After blocking using 5% nonfat milk, the Abcam primary antibodies were used, including anti-Aggrecan (ab194594, 1:1000 dilution), anti-Collagen II (ab185430, 1:2000 dilution), anti-MMP-13 (ab1010, 1:2000 dilution), anti-MMP-9 (ab119906, 1:2000 dilution), anti-CXCL12 (ab18919, 1:1000 dilution) or anti-β-actin (ab8227, 1:5000 dilution) and corresponding secondary antibody (ab6721, ab6728, 1:10000 dilution). The signals were developed using BeyoECL Plus (Beyotime) and then analyzed via Image Lab software (Bio-Rad).

### Statistical analysis

GraphPad Prism 6 software (GraphPad Inc., La Jolla, CA, USA) was used for statistical analysis. The results of 3 repeats were shown as mean ± standard deviation (S.D.). The linear correlations among HOTAIR, miR-107 and CXCL12 in OA cartilage were investigated by Spearman’s correlation analysis. Comparisons of two groups or multiple groups were performed using Student’s *t* test or one-way ANOVA. *P* value < 0.05 was considered statistically significant.

## Results

### The expression of HOTAIR is upregulated, and miR-107 level is downregulated in OA cartilage

**Fig. 1:**
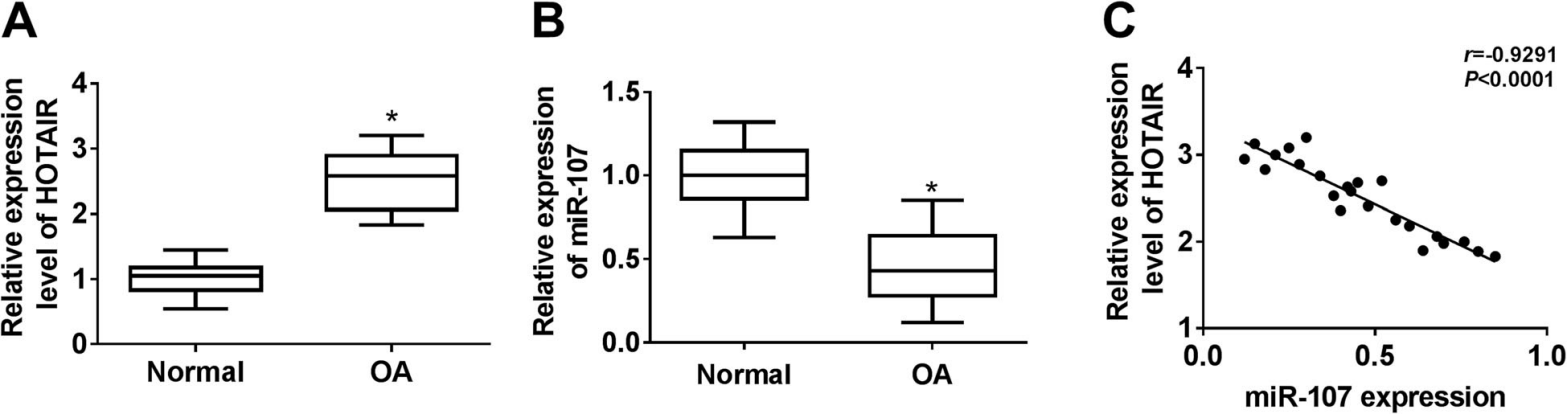
The expression of HOTAIR and miR-107 in OA. **A** and **B** The expression levels of HOTAIR and miR-107 were measured in OA and normal tissues by qRT-PCR. **C** The correlation of abundance of HOTAIR and miR-107 in OA tissues was investigated by Spearman’s correlation analysis. n = 23. *P < 0.05

To explore the role of HOTAIR and miR-107 in OA progression, their expression levels were measured in 23 OA cartilage tissues and normal samples. As shown in Fig. 1A, the expression of HOTAIR was significantly elevated in OA tissues compared with that in the normal group. Moreover, miR-107 level was obviously lower in OA cartilage than that in normal samples (Fig. 1B). Meanwhile, the expression of miR-107 in OA cartilage tissues had a negative correlation with HOTAIR level (r = − 0.9291, *P* < 0.0001) (Fig. 1C). These findings suggested that HOTAIR and miR-107 might play important roles in OA progression.

### HOTAIR regulates chondrocyte proliferation, apoptosis and ECM degradation

**Fig. 2:**
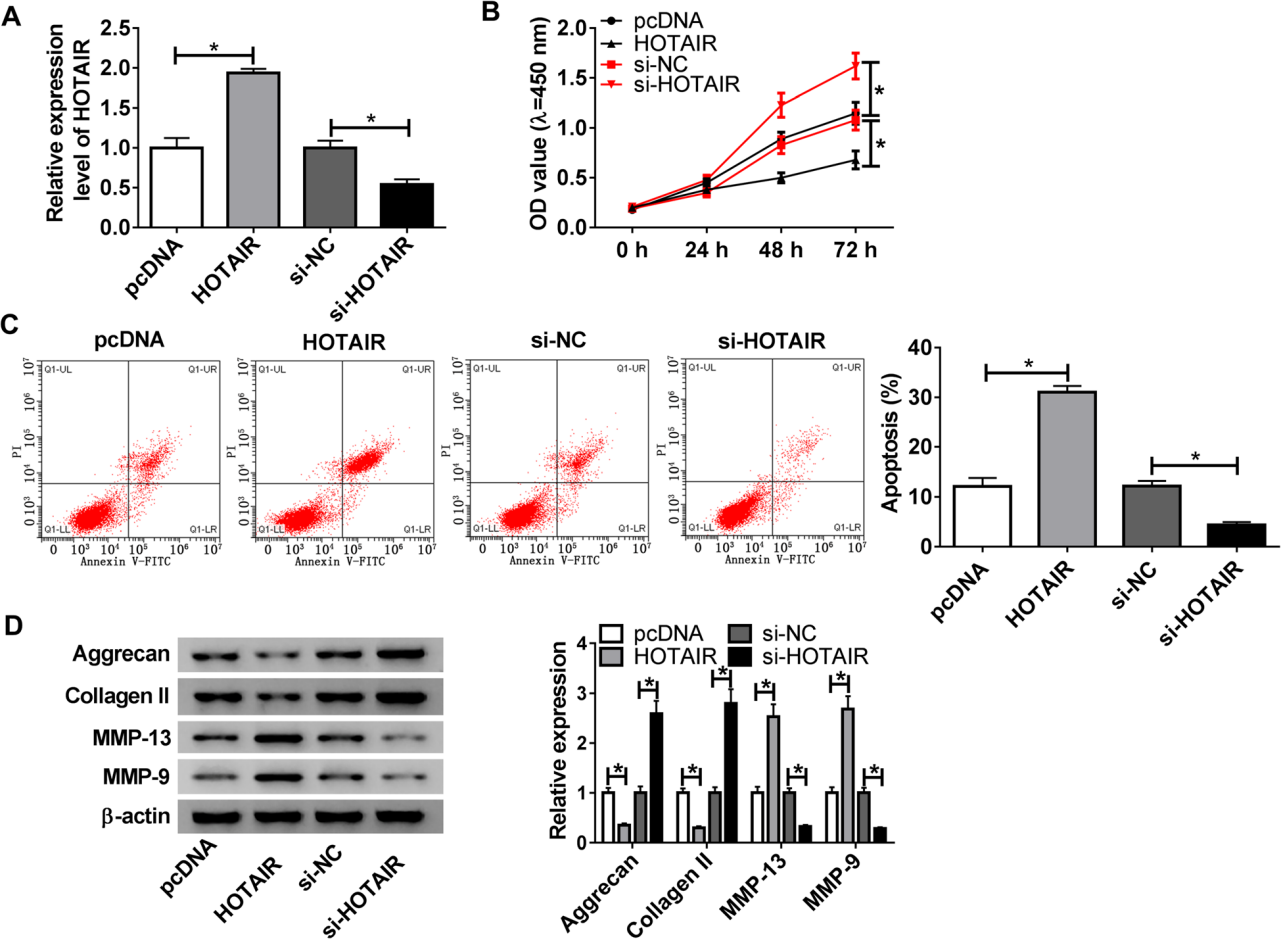
HOTAIR inhibits chondrocyte proliferation but promotes apoptosis and ECM degradation. **A** The expression of HOTAIR was detected by qRT-PCR in chondrocytes transfected with pcDNA, HOTAIR overexpression vector, si-NC or si-HOTAIR after 24-h post-transfection. **B** Cell proliferation was measured in chondrocytes transfected with pcDNA, HOTAIR overexpression vector, si-NC or si-HOTAIR by CCK-8 at 0, 24, 48 or 72 h. **C** Cell apoptosis was detected in chondrocytes transfected with pcDNA, HOTAIR overexpression vector, si-NC or si-HOTAIR by flow cytometry at 72 h after culture. **D** The protein levels of Aggrecan, Collagen II, MMP-13 and MMP-9 were detected in chondrocytes transfected with pcDNA, HOTAIR overexpression vector, si-NC or si-HOTAIR by western blot at 72 h after culture. n = 3. Data were expressed as mean ± S.D. *P < 0.05

To investigate the biological role of HOTAIR *in vitro*, its expression in chondrocytes from OA patients was upregulated by transfection of HOTAIR overexpression vector and downregulated using siRNA, which was validated by qRT-PCR (Fig. 2A). The results of CCK-8 displayed that after the culture of 72 h, chondrocyte proliferation was significantly decreased by overexpression of HOTAIR, but promoted by HOTAIR knockdown in comparison with their corresponding controls (Fig. 2B). In addition, the data of flow cytometry revealed in Fig. 2C that addition of HOTAIR induced great increase of apoptotic rate of chondrocytes, whereas silencing HOTAIR led to reduction of cell apoptosis. Moreover, HOTAIR overexpression promoted the protein expression of MMP-13 and MMP-9, but decreased the levels of Aggrecan and Collagen II in chondrocytes (Fig. 2D). However, HOTAIR knockdown led to an opposite effect. These results indicated that HOTAIR knockdown increased cell proliferation, but suppressed chondrocyte apoptosis and ECM degradation.

### HOTAIR regulates chondrocyte proliferation, apoptosis and ECM degradation by sponging miR-107

**Fig. 3:**
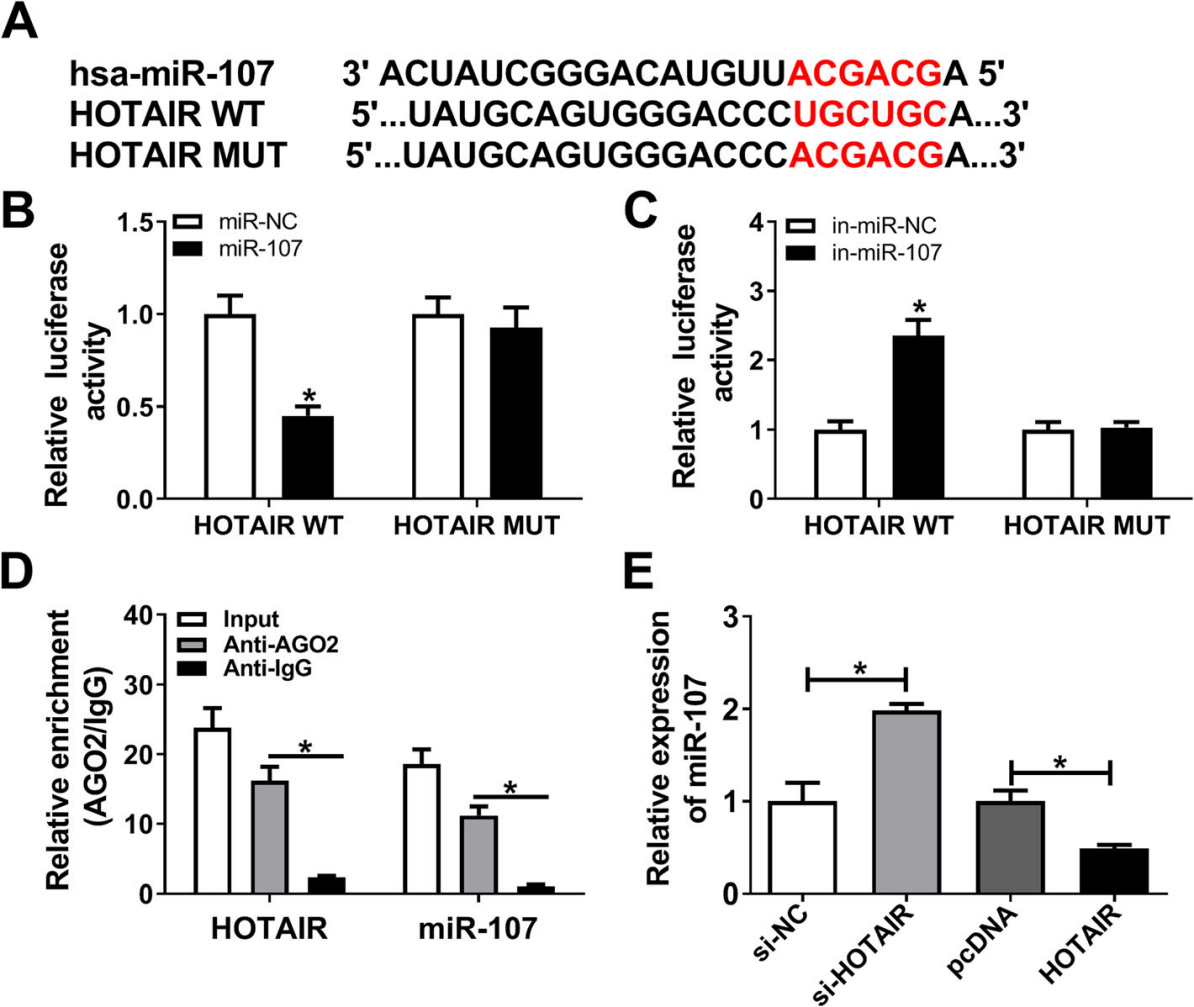
HOTAIR is a decoy of miR-107. **A** The binding sites of HOTAIR and miR-107 were predicted via DIANA tools. **B** and **C** Luciferase activity was measured in chondrocytes co-transfected with HOTAIR WT or HOTAIR MUT and miR-NC, miR-107 mimic, in-miR-NC, or in-miR-107 at 24 h post-transfection. **D** The levels of HOTAIR and miR-107 were measured after enrichment of Ago2 or IgG RIP for 8 h by qRT-PCR. **E** The expression of miR-107 was detected in chondrocytes transfected with si-NC, si-HOTAIR, pcDNA or HOTAIR overexpression vector by qRT-PCR at 24 h post-transfection. n = 3. Data were expressed as mean ± S.D. *P < 0.05

To explore how HOTAIR affected OA progression, its potential targets were explored by bioinformatics analysis using DIANA tools. We selected 5 downregulated targets (miR-107, miR-197-3p, miR-136-5p, miR-211-5p and miR-17-5p) in OA according to previous reports [17, 23–26]. Furthermore, miR-107 expression was decreased most by HOTAIR overexpression than other 4 miRNAs (Supplementary Figure 1A). Hence, miR-107 was selected for further study. As shown in Fig. 3A, the potential binding sites of HOTAIR and miR-107 were predicted, suggesting that miR-107 might be bound to HOTAIR. To prove this prediction, HOTAIR WT or HOTAIR MUT luciferase reporter vectors were generated, and transfected into chondrocytes. Results showed that overexpression of miR-107 notably reduced the luciferase activity in cells transfected with HOTAIR WT vectors, while its deficiency showed an opposite effect (Fig. 3B, C). However, overexpressing or knocking down miR-107 could not affect the luciferase activity in the HOTAIR MUT group. Moreover, the data of RIP assay revealed that HOTAIR and miR-107 were enriched by Ago2 RIP compared with that in the IgG group (Fig. 3D). Additionally, miR-107 expression level was increased by silencing HOTAIR, and decreased by HOTAIR overexpression in chondrocytes (Fig. 3E). These results suggested HOTAIR as a sponge of miR-107 in chondrocytes.

**Fig. 4:**
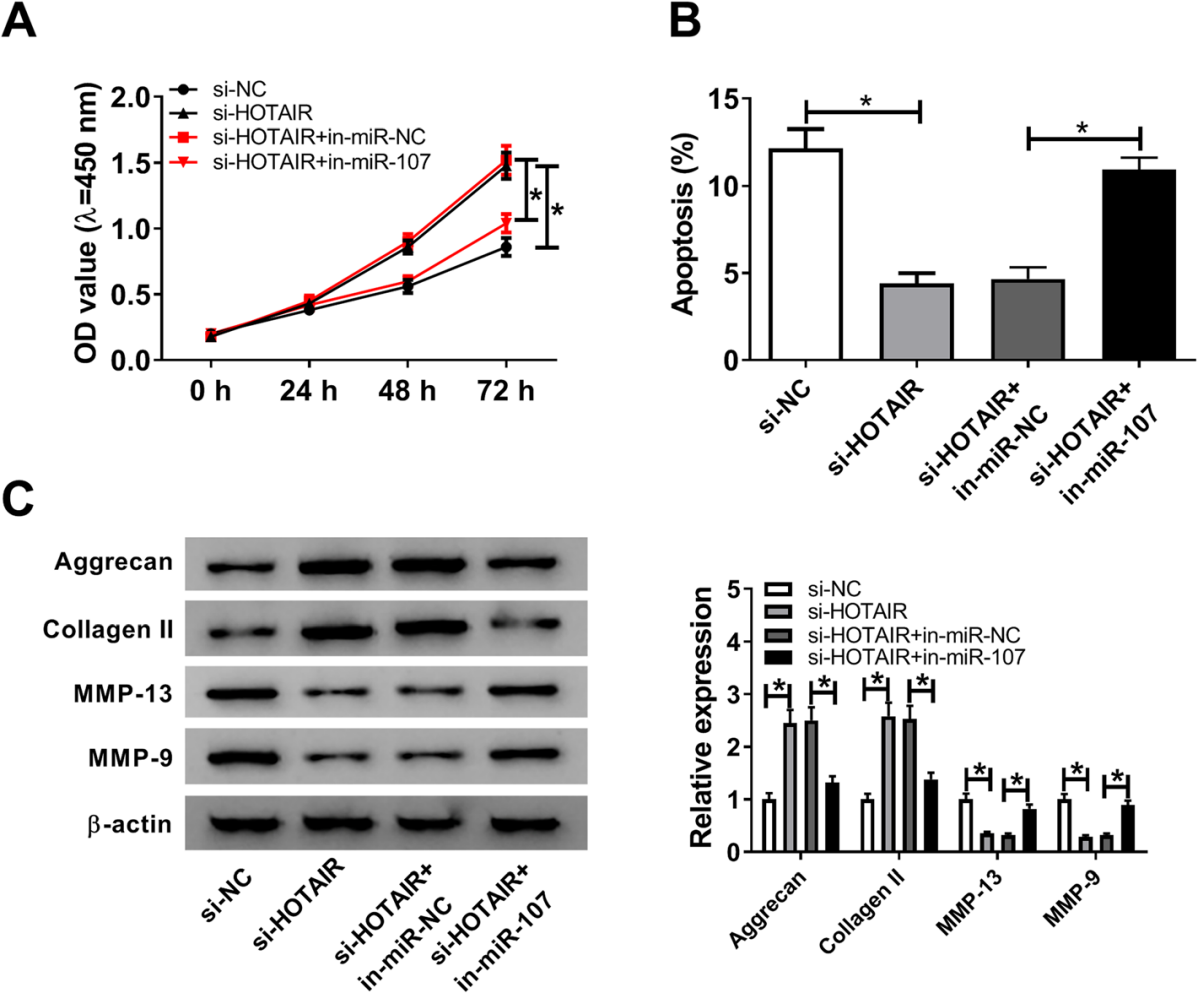
Deficiency of miR-107 reverses silencing HOTAIR-mediated chondrocyte progression. **A** Cell proliferation was measured in chondrocytes transfected with si-NC, si-HOTAIR, si-HOTAIR + in-miR-NC or in-miR-107 by CCK-8 at 0, 24, 48 or 72 h. **B** Cell apoptosis was detected in chondrocytes transfected with si-NC, si-HOTAIR, si-HOTAIR + in-miR-NC or in-miR-107 by flow cytometry at 72 h after culture. **C** The protein levels of Aggrecan, Collagen II, MMP-13 and MMP-9 were examined in chondrocytes transfected with si-NC, si-HOTAIR, si-HOTAIR + in-miR-NC or in-miR-107 by western blot at 72 h after culture. n = 3. Data were expressed as mean ± S.D. *P < 0.05

To investigate whether miR-107 was required for HOTAIR-mediated OA progression, chondrocytes were transfected with si-NC, si-HOTAIR, si-HOTAIR + in-miR-NC or in-miR-107, followed by investigation of cell function. As demonstrated in Fig. 4A, miR-107 exhaustion abrogated silencing HOTAIR-mediated promotion of proliferation in chondrocytes. Furthermore, knockdown of miR-107 attenuated downregulation of HOTAIR-mediated apoptosis inhibition in chondrocytes (Fig. 4B). Besides, inhibition of miR-107 weakened knockdown of HOTAIR-mediated upregulation of Aggrecan and Collagen II as well as downregulation of MMP-13 and MMP-9 (Fig. 4C). These findings indicated that HOTAIR mediated chondrocyte injury relying on the regulation of miR-107.

### HOTAIR regulates CXCL12 expression to mediate chondrocyte progression by sponging miR-107

**Fig. 5:**
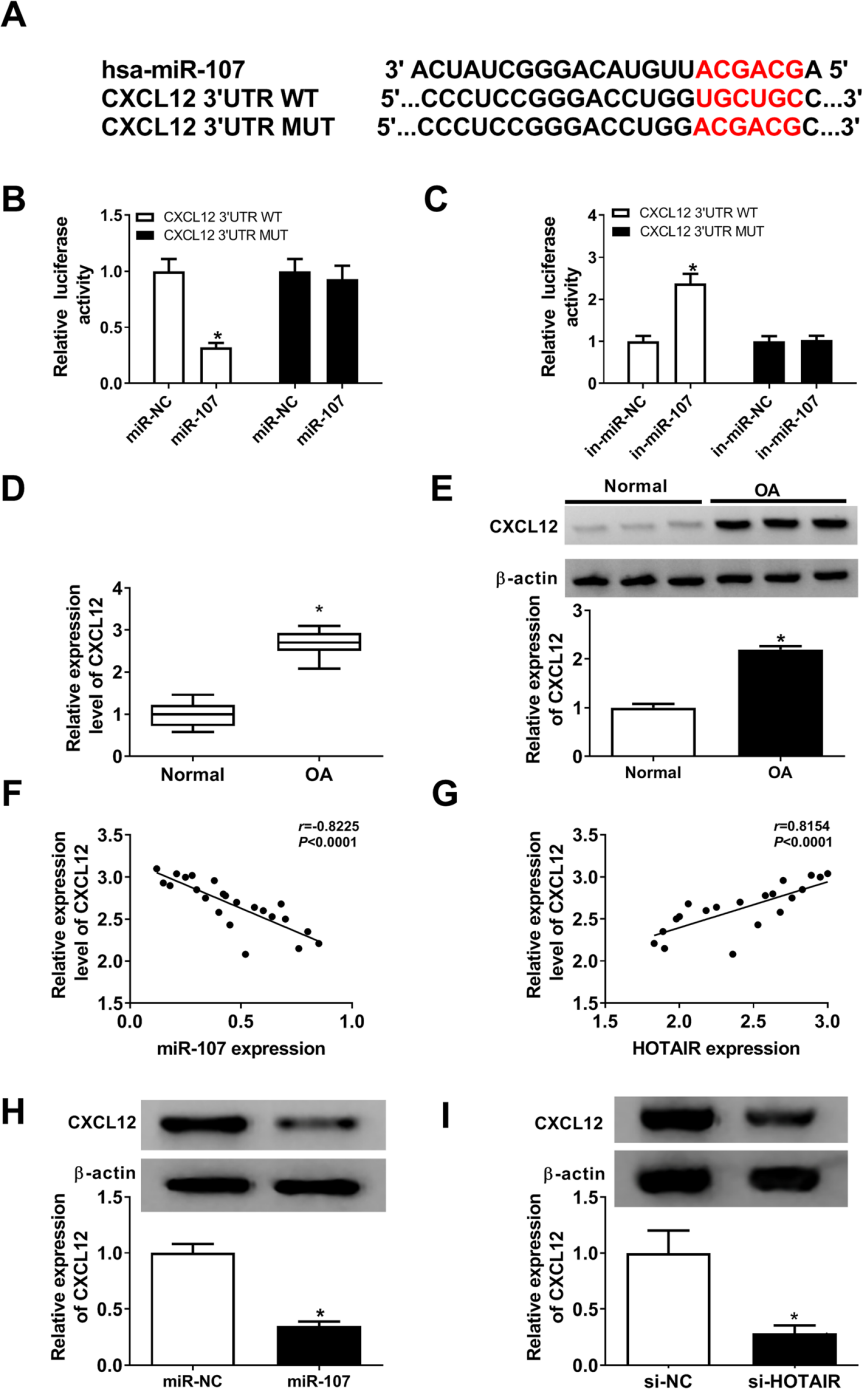
CXCL12 is a target of miR-107. **A** The binding sites of miR-107 and CXCL12 were predicted via DIANA tools. **B** and **C** Luciferase activity was measured in chondrocytes co-transfected with CXCL12 3’UTR WT or CXCL12 3’UTR MUT and miR-NC, miR-107 mimic, in-miR-NC or in-miR-107 at 24 h post-transfection. **D** and **E** The expression of CXCL12 at mRNA and protein levels was measured in OA and normal samples by qRT-PCR and western blot. **F** and **G** The correlation of abundance of CXCL12 and miR-107 or HOTAIR in OA tissues was evaluated by Spearman’s correlation analysis. **H** The protein level of CXCL12 was measured in chondrocytes transfected with miR-NC or miR-107 mimic by western blot at 24 h post-transfection. **I** The protein level of CXCL12 was detected in chondrocytes transfected with si-NC or si-HOTAIR by western blot at 24 h post-transfection. n = 3. Data were expressed as mean ± S.D. *P < 0.05

To further explore the mechanism in this study, bioinformatics analysis was performed using DIANA tools. Six upregulated targets (CXCL12, FUT2, FUT1, EIF4G2, EZH2 and PTEN) of miR-107 were selected according to previous reports [14, 16, 20, 23, 27, 28]. Moreover, CXCL12 abundance was reduced most via miR-107 overexpression than other 5 mRNAs (Supplementary Figure 1B). Hence, CSCL12 was selected for further study. The potential binding sites of miR-107 and CXCL12 are shown in Fig. 5A. The luciferase reporter assay demonstrated that CXCL12 was a target of miR-107, revealed by effect of miR-107 on luciferase activity (Fig. 5B, C). Furthermore, the expression of CXCL12 was measured in OA and normal samples (n = 23). As described in Figs. 5D and E, the mRNA and protein levels of CXCL12 were abnormally enhanced in OA cartilage compared with that in the normal group. Meanwhile, the expression of CXCL12 in OA cartilage was negatively associated with miR-107 level (r = − 0.8225, *P* < 0.0001) (Fig. 5F), but positively correlated with HOTAIR abundance (r = 0.8154, *P* < 0.0001) (Fig. 5G). In addition, the expression of CXCL12 protein was negatively regulated by miR-107 overexpression or HOTAIR interference (Fig. 5H, I).

**Fig. 6:**
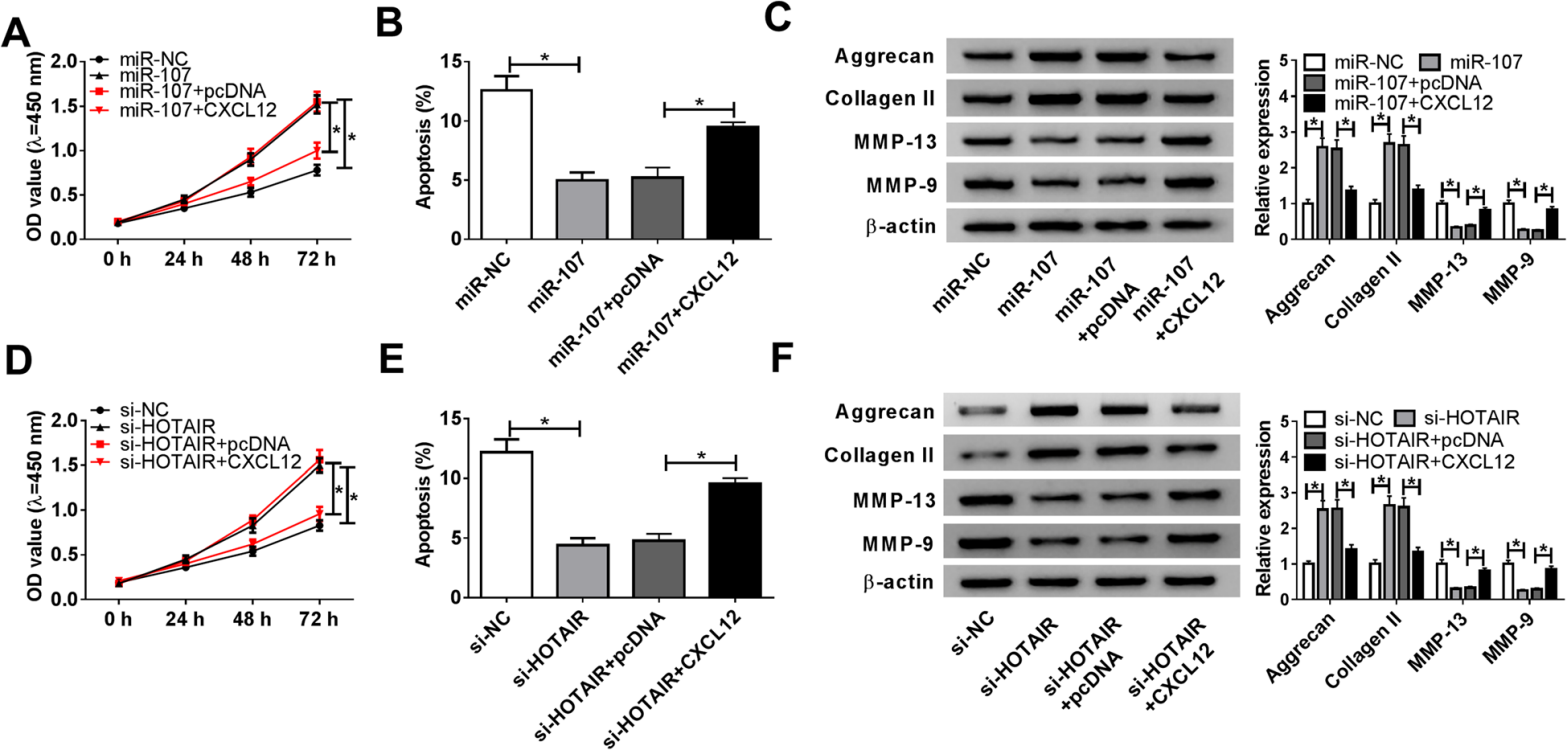
CXCL12 overexpression alleviates miR-107 or silencing HOTAIR-mediated chondrocyte progression. **A** Cell proliferation was measured in chondrocytes transfected with miR-NC, miR-107 mimic, miR-107 mimic + pcDNA or CXCL12 overexpression vector by CCK-8 at 0, 24, 48 or 72 h. **B** Cell apoptosis was detected in chondrocytes transfected with miR-NC, miR-107 mimic, miR-107 mimic + pcDNA or CXCL12 overexpression vector by flow cytometry at 72 h after culture. **C** The protein levels of Aggrecan, Collagen II, MMP-13 and MMP-9 were measured in chondrocytes transfected with miR-NC, miR-107 mimic, miR-107 mimic + pcDNA or CXCL12 overexpression vector by western blot at 72 h after culture. **D** Cell proliferation was measured in chondrocytes transfected with si-NC, si-HOTAIR, si-HOTAIR + pcDNA or CXCL12 overexpression vector by CCK-8 at 0, 24, 48 or 72 h. **E** Cell apoptosis was detected in chondrocytes transfected with si-NC, si-HOTAIR, si-HOTAIR + pcDNA or CXCL12 overexpression vector by flow cytometry at 72 h after culture. **F** The protein levels of Aggrecan, Collagen II, MMP-13 and MMP-9 were measured in chondrocytes transfected with si-NC, si-HOTAIR, si-HOTAIR + pcDNA or CXCL12 overexpression vector by western blot at 72 h after culture. n = 3. Data were expressed as mean ± S.D. *P < 0.05

To investigate whether CXCL12 was involved in the HOTAIR-mediated mechanism, its abundance was overexpressed in chondrocytes using the overexpression vector on the basis of miR-107 overexpression or HOTAIR silence. As shown in Figs. 6A and B, overexpression of miR-107 promoted chondrocyte proliferation and inhibited apoptosis. Moreover, miR-107 resulted in the upregulation of Aggrecan and Collagen II as well as the downregulation of MMP-13 and MMP-9 in chondrocytes (Fig. 6C). Additionally, these events were weakened by restoration of CXCL12 (Fig. 6A–C). Besides, overexpression of CXCL12 evidently overturned the regulatory role of HOTAIR knockdown in proliferation, apoptosis and ECM-related protein expression in chondrocytes (Fig. 6D–F).

**Fig. 7:**
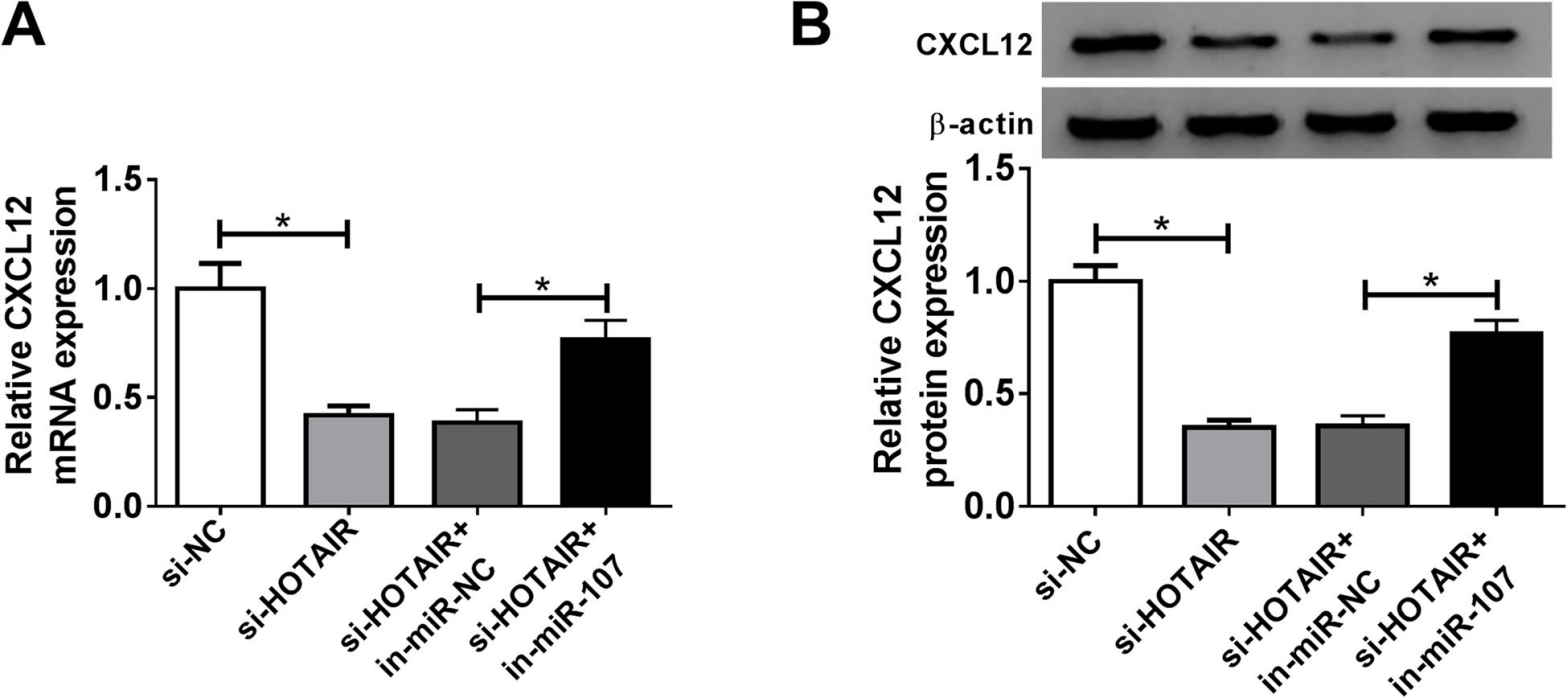
HOTAIR regulates CXCL12 expression by competitively sponging miR-107. **A** and **B** The abundances of CXCL12 at mRNA and protein levels were measured in chondrocytes transfected with si-NC, si-HOTAIR, si-HOTAIR + in-miR-NC or in-miR-107 by qRT-PCR and western blot at 24 h post-transfection. n = 3. Data were expressed as mean ± S.D. *P < 0.05

Moreover, the results of qRT-PCR and western blot exhibited that CXCL12 mRNA and protein levels in chondrocytes were significantly reduced by HOTAIR silence, while this event was attenuated by exhaustion of miR-107 (Fig. 7A, B). These data reflected that HOTAIR regulated chondrocyte progression by sponging miR-107 and regulating CXCL12.

## Discussion

LncRNAs play important roles in chondrogenesis and OA progression via mediating inflammatory response, ECM degradation and apoptosis [29, 30]. This work revealed that HOTAIR expression was enhanced in OA tissues, suggesting that HOTAIR might contribute to OA progression, which is also in agreement with the previous study [31]. In this work, we focused on the role and mechanism of HOTAIR in the development of OA. Here we first demonstrated that HOTAIR knockdown inhibited chondrocyte injury by regulating miR-107/CXCL12.

A previous report suggested that HOTAIR might promote chondrocyte apoptosis and matrix metalloproteinase (MMP) expression [32]. Moreover, HOTAIR could induce cartilage degradation by activating the Wnt pathway [33]. Additionally, HOTAIR could promote chondrocyte apoptosis and ECM degradation by regulating miR-20b or miR-130a-3p in OA [34, 35]. This study described that HOTAIR suppressed chondrocyte proliferation and promoted apoptosis, which is induced by the pro-apoptotic role of HOTAIR. Moreover, the structure of ECM is required for the normal function of cartilage, and ECM degradation of chondrocytes contributes to the development of OA [36, 37]. Aggrecan and Collagen II are the constituents of ECM [38]. The degradative matrix metalloproteinases (such as MMP-13 and MMP-9) are responsible for the degradation of Aggrecan and Collagen II proteins, which contribute to ECM degradation during OA pathogenesis [39, 40]. By detecting these protein expression, we found that HOTAIR promoted MMP-13 and MMP-9 expression but inhibited Aggrecan and Collagen II levels, indicating that HOTAIR promoted ECM degradation, which was also in agreement with the previous study [14]. Meanwhile, ECM degradation was also repressed by the knockdown of HOTAIR. These findings indicated that HOTAIR knockdown might protect against chondrocyte injury by inhibiting cell apoptosis and ECM degradation, leading to the inhibition of OA progression. However, the molecular mechanism by which HOTAIR mediates OA progression through the miRNA/mRNA axis remains unclear.

The ceRNA network is the main mechanism that allows lncRNA participating in cancer or disease progression. HOTAIR has been revealed to regulate cell proliferation, migration and invasion via functioning as a ceRNA for miRNAs, such as miR-126-5p and miR-646 [41, 42]. Moreover, it was reported that HOTAIR could regulate miR-17-5p/FUT2 or miR-20b/PTEN axis to promote chondrocyte damage during OA [14, 34]. Our study wanted to explore an additional regulatory network mediated by this lncRNA. Here we firstly confirmed miR-107 was targeted by HOTAIR. miR-107 expression is declined in OA cartilage, and its overexpression promoted cell proliferation but suppressed apoptosis and ECM degradation, suggesting the protecting role of miR-107 in OA chondrocytes, which was consistent with a former research [17]. The negative correlation between HOTAIR and miR-107 expression levels stimulated our interest to analyze whether HOTAIR regulated miR-107 on a ceRNA-based mechanism. This study demonstrated that HOTAIR is a decoy of miR-107, which was validated by the luciferase reporter assay and RIP. In addition, knockdown of miR-107 abrogated silencing HOTAIR-mediated inhibition of chondrocyte injury, indicating that HOTAIR regulated OA progression by sponging miR-107.

To further elucidate the ceRNA network, the target of miR-107 was explored. This study first confirmed that CXCL12 was targeted by miR-107 using the luciferase reporter assay. CXCL12 could mediate CXCR4 to activate inflammatory signaling to induce chondrocyte apoptosis or promote the secretion of MMPs to degrade the surrounding ECM [43–45]. Previous studies reported that CXCL12 could promote OA progression by decreasing chondrocyte proliferation and promoting ECM degradation [27, 46]. In this study, our findings exhibited that CXCL12 mitigated the effect of miR-107 overexpression and HOTAIR knockdown on chondrocyte proliferation, apoptosis and ECM degradation, indicating the important role of CXCL12 in OA progression. Furthermore, we found that knockdown of HOTAIR decreased CXCL12 expression in chondrocytes. However, this effect was attenuated by downregulation of miR-107, indicating that HOTAIR regulated CXCL12 expression by modulating miR-107.

However, there were some limitations in our study. Previous studies reported that the chondrocytes treated via IL-1β, TNF-α or LPS were widely used to mimic the OA model [47, 48], and some studies have used U937-activated monocyte-conditioned medium to stimulate OA chondrocytes to induce OA [49]. Our research uses untreated OA chondrocytes for research, which may not completely mimic the inflammatory environment of OA. In future studies, we will simulate the inflammatory environment of OA *in vitro* to further confirm our conclusions. Additionally, the upstream and downstream effectors of HOTAIR/miR-107/CXCL12 in OA progression were not explored in this study, which should be explored in further study. Furthermore, the *in vivo* experimental models were absent in the current research. Hence, an animal model of OA should be established to further investigate this network in future.

In short, HOTAIR was highly expressed in OA cartilage, and its knockdown inhibited the dysfunction of chondrocytes by promoting chondrocyte proliferation and suppressing apoptosis and ECM degradation. We further confirmed this effect might require miR-107/CXCL12 axis. This elucidates a new mechanism underlying chondrocyte damage, which might indicate a novel insight into OA progression and provide novel target for the treatment of OA.

## Supplementary Information

**Additional file 1 Supplementary Figure 1 The effect of HOTAIR or miR-107 on downstream target levels.** (A) MiR-107, miR-197-3p, miR-136-5p, miR-211-5p and miR-17-5p levels were detected in chondrocytes transfected with pcDNA or HOTAIR overexpression vector at 24 h post-transfection. (B) CXCL12, FUT2, FUT1, EIF4G2, EZH2 and PTEN levels were measured in chondrocytes transfected with miR-NC or miR-107 mimic at 24 h post-transfection. n=3. Data were expressed as mean ± S.D. *P<0.05.

## Data Availability

The analyzed data sets generated during the present study are available from the corresponding author on reasonable request.
