# Supplementary figures and images for: Knockdown of long noncoding RNA HOTAIR inhibits osteoarthritis chondrocyte injury by miR-107/CXCL12 axis
Source: J Orthop Surg Res. 2021 Jun 28;16:410. doi: 10.1186/s13018-021-02547-7 (PMC8237457; doi:10.1186/s13018-021-02547-7)

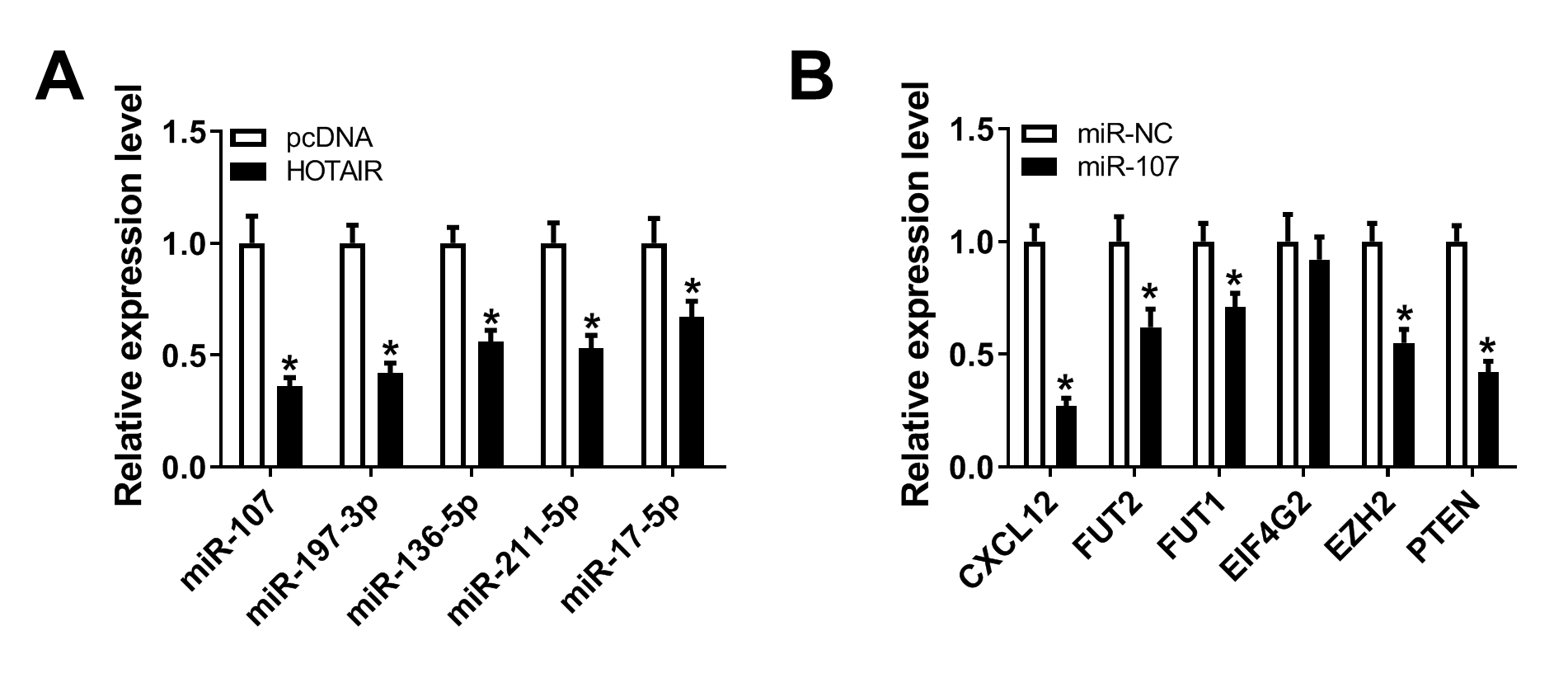

Supplement: Supplementary file 1 — Additional file 1 Supplementary Figure 1 The effect of HOTAIR or miR-107 on downstream target levels. (A) MiR-107, miR-197-3p, miR-136-5p, miR-211-5p and miR-17-5p levels were detected in chondrocytes transfected with pcDNA or HOTAIR overexpression vector at 24 h post-transfection. (B) CXCL12, FUT2, FUT1, EIF4G2, EZH2 and PTEN levels were measured in chondrocytes transfected with miR-NC or miR-107 mimic at 24 h post-transfection. n=3. Data were expressed as mean ± S.D. *P<0.05. [file 13018_2021_2547_MOESM1_ESM.tif]
